# Decrease of left ventricular ejection fraction in severe illness patients due to COVID-19 may improve as the disease resolves

**DOI:** 10.3906/sag-2101-198

**Published:** 2021-09-07

**Authors:** Aykut DEMİRKIRAN, Lütfi Çağatay ONAR, Mustafa DOĞAN

**Affiliations:** 1 Department of Cardiology, Tekirdağ Dr. İsmail Fehmi Cumalıoğlu City Hospital, Tekirdağ, Turkey; 2 Department of Cardiovascular Surgery, Tekirdağ Dr. İsmail Fehmi Cumalıoğlu City Hospital, Tekirdağ, Turkey; 3 Department of Infectious Diseases and Clinical Microbiology, Tekirdağ Namık Kemal University, Tekirdağ, Turkey

**Keywords:** COVID-19, left ventricular ejection fraction, myocardial dysfunction

## Abstract

**Background/aim:**

Increase in publications supporting myocardial involvement in the COVID-19 disease has led to need to gain insight into the the global burden of heart failure after pandemic. We examined the course of myocardial systolic function in patients without elevated troponin levels.

**Materials and methods:**

We performed a prospective study. Patients with high troponin levels were excluded from the study in order to definitively exclude complications known to cause permanent left ventricular systolic dysfunction, such as acute coronary syndromes. Two echocardiographic examinations were performed. The first evaluation was performed within the days of hospitalization, if possible, on the day when dyspnea is severe. The second evaluation was performed during the outpatient clinic controls one month after the patient was recovered. Left ventricular ejection fraction (LVEF) was measured using the biplane method of disks (modified Simpson’s rule).

**Results:**

In the first evaluation, LVEF was found to be significantly lower in the severe illness group than mild/moderate illness group (50 ± 6% and 59 ± 6%; p = 0.03). LVEF decrease (<50%) was found in fifteen patients (43 ± 4%) and detected as global hypokinesia but not segmental. All of these patients were in the severe illness group. In the second evaluation, LVEFs of the fifteen patients with decreased LVEF in the first evaluation were improved and detected in normal limits (first evaluation = 43 ± 4% and second evaluation = 55 ± 2%, p = 0.01).

**Conclusion:**

Considering patients without elevated troponin levels during COVID-19 infection, no permanent systolic dysfunction was detected after first month of recovery. We found that transient myocardial dysfunction may develop in the severe illness group with normal troponin levels, LVEF may decrease in the acute phase and improve with the recovery period.

## 1. Introduction

Coronavirus Disease 2019 (COVID-19) has become a global pandemic. In addition to systemic and respiratory complications, COVID-19 can affect the cardiovascular system. Cardiovascular interactions may occur due to acute coronary syndrome, demand ischemia, microvascular ischemic injury, injury related to cytokine dysregulation or myocarditis [1]. Patterns of troponin release, myocarditis or cytokine/stress-related cardiomyopathy due to the COVID-19 are not well defined. Myocarditis, systemic cytokine-mediated, stress-related cardiomyopathy, or microvascular thrombosis could produce myocardial injury. Within a few days of myocardial injury, edema and necrosis can lead to contractile dysfunction [2,3]. All cardiologists must be aware of the potential impact that this disease can cause in their respective fields. In the light of recent studies, although the mechanism of myocardial damage due to COVID-19 is not fully comprehended, SARS-CoV-2 enters human cells by binding to angiotensin-converting enzyme 2 (ACE2), highly expressed in the heart. The binding to ACE2 can lead to acute myocardial injury [4]. More severe forms of COVID-19 are characterized by acute systemic inflammatory response and cytokine storm, which can result in injury to multiple organs leading to multiorgan failure [5]. It can be thought that as the severity of the disease increases, cytokine release will increase. Adults with COVID-19 infection were grouped into the following severity of illness categories according to the guidelines (National Institutes of Health. Coronavirus Disease 2019 (COVID-19) Treatment Guidelines. https://www.covid19treatmentguidelines.nih.gov/, accessed 1/11/2020): asymptomatic infection, mild illness (individuals who have any of the various signs and symptoms of COVID-19 but who do not have shortness of breath, dyspnea, or abnormal chest imaging), moderate illness (individuals who show evidence of lower respiratory disease during clinical assessment or imaging and who have a saturation of oxygen (SpO2) ≥ 94% on room air at sea level), severe illness (individuals who have SpO2 < 94% on room air at sea level, a ratio of arterial partial pressure of oxygen to fraction of inspired oxygen (PaO2 / FiO2) < 300 mmHg, respiratory frequency > 30 breaths / min, or lung infiltrates > 50%), critical illness (individuals who have respiratory failure, septic shock, and/or multiple organ dysfunction). Studies have shown high circulatory levels of proinflammatory cytokines in patients with severe/critical COVID-19 [6].

Left ventricular ejection fraction (LVEF) is the measure of left ventricular systolic function. It is important to know the course of heart failure in COVID-19. We planned this study to contribute to the understanding of the cardiac effects of COVID-19. In this study, we examined the course of myocardial systolic function and whether there is a permanent myocardial contractile dysfunction.

## 2. Materials and methods

### 2.1. Study design

We performed a prospective study at a government-authorized hospital for COVID-19 patients. The diagnosis of COVID-19 was confirmed as a positive result for nasopharyngeal nucleic acid test with real-time (RT) reverse transcriptase-polymerase chain reaction (PCR) for COVID-19. RT-PCR tests were performed with the BIO-RAD Touch Real-Time PCR Detection System (Model no. CFX96 Optics Module, serial number: 785BR25673) in the presence of a commercial kit (Diagnovital HS SARS-Cov-2 Multiplex Real-Time PCR Kit, Cat. 11-014, A1 Life Sciences, İstanbul, Turkey). In order to follow-up more closely shortness of breath, only patients who were hospitalized were included in our prospective study.

### 2.2. Study sample

All patients were enrolled with a diagnosis of COVID-19 from April 1 to November 20, 2020. Patients above 18 years old and PCR positive were included in the study. Patients with PCR negative were excluded. Patients who had previously undergone echocardiography and were found to have decreased LVEF were also excluded from the study because it would complicate the evaluation. Image quality is important in echocardiographic evaluation for our study. Patients who required mechanical ventilation were not included in the study, because the echocardiographic examination in intubated patients may be suboptimal.

One of the most important markers of myocardial damage is elevation of cardiac troponins. Troponins may be elevated in acute coronary syndromes and myocarditis occurring during the course of COVID-19. Decreased left ventricular systolic functions may be detected in these patients. In the follow-up of patients in our clinics, we found that cardiac functions decreased in patients whose troponin levels were not high during the course of COVID-19. Therefore, we predicted that cardiac troponin elevation was not the only criterion of myocardial dysfunction. Patients with high troponin levels ( > 18ug / L) were excluded to prove our hypothesis.

The patients were divided into two groups according to the severity of the illness specified in the current guidelines: severe illness and mild/moderate illness group.

### 2.3. Echocardiographic examination

Two echocardiography and ECG procedures were performed for each patient. The first evaluation was performed within the days of hospitalization. Patients without dyspnea were evaluated randomly on any day of their hospitalization process. If dyspnea developed during the hospital follow-up, the first evaluation was performed when the patient had active dyspnea. Patients were called for control 30 days after they were discharged from the hospital. The second evaluation was performed during the outpatient clinic controls.

Echocardiographic examination was performed using a Philips EPIQ 7 Cardiology Ultrasound Machine with an S5-1 transducer (Philips N.V.,Amsterdam, Netherlands). Parasternal and apical views were recorded. All datasets were stored on a workstation for offline analysis (EPIQ QLAB Automated Cardiac Motion Quantification software) by a cardiologist blinded to the clinical data. For each measurement, at least three cardiac cycles were averaged. LV end-diastolic and end-systolic diameters and LVEF were measured using the biplane method of disks.

### 2.4. Ethical considerations

Our study was approved by the Local Ethics Committee (2020.140.06.02). Patients were clarified of the study’s purpose and explained that they could leave from the study at any time.

### 2.5. Statistical analysis

All of the statistical analyses were performed with the IBM SPSS statistics for windows, version 23.0 software package (IBM Corp., Armonk, NY). The distribution of continuous variables was evaluated using histograms, normal Q–Q plots, skewness, kurtosis, and Kolmogorov–Smirnov test. The frequency analysis and descriptive statistics was used to describe data. The independent samples t-test was used for normally distributed data and the Mann–Whitney U test for non-normally distributed data. Normally distributed echocardiographic parameters in consecutive echocardiograms were compared with the paired t test. Non-normally distributed data in consecutive echocardiograms were analyzed by use of the signed Wilcoxon rank-sum test. Values of p < 0.05 were considered to indicate statistical significance.

## 3. Results

### 3.1. Demographic and clinical characteristic

A total of 160 patients were enrolled. But 3 patients died after the first examination, ST-elevation myocardial infarction occurred in 3 patients after the first examination, and 4 patients had poor image quality. The remaining 150 patients were studied. The baseline clinical characteristics of patients were summarized in Table 1. The median age was 46 ± 5 years, 52% (78) of patients were male and, the mean body mass index (BMI) was 30 ± 4 kg/m2. The most common symptom was cough (74%). 20% of the patients had developed dyspnea. Fifty (33%) patients who developed low saturation (SpO2 < 94% on room air) were transferred to ICU. Although twenty patients did not describe shortness of breath, hypoxia was diagnosed. On questioning, they consistently denied any difficulty with breathing. No respiratory failure or shock developed in any of the patients during the follow-up.

### 3.2. Cardiac imaging findings

#### 3.2.1. First evaluation

Left ventricular ejection fraction measurement was found to be under 50% in fifteen (10%) patients and were found to be under 40% in two (1%) patients. Left ventricular wall motion abnormalities were detected as mild global hypokinesis, but not segmental or at a specific location. Thirty (20%) patients have presented the signs of pulmonary hypertension (peak tricuspid regurgitation velocity > 3 m / s). Pericardial effusion was not detected in any patient. All patients were in sinus rhythm, and the mean heart rate was 95 ± 10 / min. The COVID-19 treatment performed on our patients is given in Table 2.

#### 3.2.2. Second evaluation

Patients were called for outpatient clinic control. None of the patients, including fifteen patients with left ventricular ejection fraction less than 50% at the initial evaluation, had dyspnea, weakness, fatigue, sore throat, muscle pain, fever. Twenty four patients described psychiatric disorders (sleep disorder, anxiety disorder). On physical examination, mean heart rate was decreased compared to the initial evaluation (95 ± 10 and 75 ± 10 beats/min, p = 0.02). There was no difference in blood pressure measurements in the first and second evaluations (systolic blood pressures 135 ± 10 and 130 ± 8 mmHg, p = 0.21, diastolic blood pressures 84 ± 4 and 80 ± 5mmHg, p = 0.30).

LVEDD and LVESD were found within normal limits in the second echocardiographic examination. The LVEFs of all patients, including fifteen patients with LVEF under 50% in first evaluation, were > 50% (60 ± 5). Systolic pulmonary artery pressure of all patients including thirty patients with pulmonary hypertension at initial evaluation were found to be < 30 mmHg. Comparison of the first and second echocardiographic findings of all patients were summarized in Table 3.

Progression of illness severity may have a potential impact on myocardial function so that the patients were divided into two groups as described in the method section: severe illness and mild/moderate illness group. The comparison of the two groups is given in Table 4. The LVEF decrease was significant in severe illness group in the first echocardiographic examination (59 ± 6 and 50 ± 6, p = 0.03).

When patients with LVEF < 50% (n = 15) are compared with other patients (n = 135), it is observed that all the patients with decreased LVEF were in the severe illness group requiring follow-up in ICU for monitoring and treatment. Mean body mass index was higher (34 ± 4 vs 30 ± 6, p = 0.02), systolic pulmonary artery pressure was increased (38 ± 8 vs 24 ± 4, p = 0.02) (Table 1). In these 15 patients, the mean LVEF was increased from 43 ± 4 to 55 ± 2 in one month after discharge (p = 0.01) (Table 5). It was found that coronary angiography was performed for 4 of 15 patients during follow-up. Critical stenosis that may cause ischemia was not detected. Example of patient is shown in Figure A–F.

## 4. Discussion

According to these results, LVEF decrease due to COVID-19 may be temporary in patients whose troponin levels are not elevated during follow-up. Unlike other severe infection situations, COVID-19 infection can reduce left ventricular systolic functions even without developing septic shock. As the severity of the illness increases, myocardial dysfunction becomes more frequent, but myocardial dysfunction may improve after the recovery. In cases whose troponin levels were not elevated during infection, we can conclude that myocardial dysfunction will improve. Necrosis or permanent myocardial dysfunction was not detected in our study cohort. Considering the number of patients without troponin elevation, improvement in cardiac functions may predict the economic and social burden of heart failure in the post-COVID-19 pandemic. Among 72,314 persons with COVID-19 in China, 81% of cases were reported to be mild, 14% were severe, and 5% were critical [7].

We found that the LVEF was slightly lower in our patients, with fifteen (10%) of patients showing a reduction of LVEF < 50% during the acute COVID-19 infection. Previous studies have revealed that other types of coronavirus including SARS-CoV can cause myocardial inflammation and damage [8]. Our study results suggest that LV systolic dysfunction occurs in patients with acute COVID-19 infection, but it is not very common. The exact pathophysiological mechanism underlying myocardial injury caused by COVID-19 is not fully understood. Systemic inflammation represents an advanced illness, characterized by multiple organ failure [9]. Studies have demonstrated that decreased contractility and impaired myocardial compliance are factors leading to myocardial dysfunction in sepsis. The number of mediators and circulatory factors (cytokines, prostanoids, and endothelin) is known to be associated with myocardial depression during sepsis [10]. COVID-19-associated sepsis is similar to bacterial sepsis and ARDS [11]. Sepsis frequently complicates COVID-19 among hospitalized patients and is significantly higher among those in the ICU. In our study, critical illness and septic shock patients were not included due to the difficulty of positioning for echocardiographic images. Our patients did not develop sepsis during hospitalization follow-up. Left ventricular systolic dysfunction may develop in patients without sepsis and septic shock according to our study results.

Characteristics of fifteen patients with transient LVEF decrease were presented in Table 5. Most clinical characteristics were similar between patients except for higher duration of complaints, body mass index, creatinine, BNP, albumin, and ferritin in patients with transient LVEF decrease. We could not measure other inflammatory markers include IL (interleukin)-6, IL-2, IL-7, TNF (tumor necrosis factor)-α, IFN (interferon)-γ IP (inducible protein)-10, MCP (monocyte chemoattractant protein)-1, MIP (macrophage inflammatory protein)-1α, G-CSF (granulocyte-colony stimulating factor) levels [12]. All of our patients with transient decreased LVEF were hospitalized in ICU due to dyspnea. In addition to the treatments described in Table 2, eleven patients required high flow nasal cannula, and four patients required non-invasive ventilation. Anticoagulant (enoxaparin 4.000 IU; 6000 UI if body weight > 100 kg) and diuretic ( 20 to 60 mg / day intravenous furosemide) were used in all fifteen patients. Two patients required immunotherapy (convalescent immune plasma of improved cases from COVID-19 disease). We used selective cytokine blockade agents in four patients (anakinra and tocilizumab). In our study, improvement of the wall motion abnormality was shown after recovery. The amount of circulating cardiac depressants may affect myocyte functions in the course of COVID-19. The decrease amount of circulating cardiac depressants with recovery may explain the improvement of the wall motion abnormality. Early initiation of agents that reduce mediators and circulatory factors release may reduce cardiac damage and accelerate recovery in LVEF.

For patients with a transient ventricular motion abnormality, possible diagnoses include stress cardiomyopathy. Diagnosis of stress cardiomyopathy is based on the identification of the following features: transient left ventricular systolic dysfunction (typically not in a single coronary distribution; patterns include apical, mid-ventricular, and basal), absence of angiographic evidence of obstructive coronary disease, and increase in troponin levels is expected. Takotsubo cardiomyopathy and neurogenic stunning myocardium share common clinical features such as reversible LV dysfunction. They also share common pathophysiology, exaggerated sympathetic stimulation, and elevated plasma catecholamines that may result in direct myocyte injury via an increase in intracellular calcium and oxygen-free radicals, diffuse coronary microvascular dysfunction, multivessel epicardial spasm, transient dynamic LV outflow tract obstruction, or direct injury to myocytes [13].

Our study has several limitations. Echocardiographic examinations were performed during the active infection period. Protecting healthcare workers from intense contact was considered; therefore, the number of examinations was kept limited in our study. It should also be kept in mind that asymptomatic patients who do not require hospitalization and patients with respiratory failure requiring intubation/ mechanical ventilation were not included in our study. It is necessary to investigate whether myocardial functions may be more depressed in critical illness.

## 5. Conclusion

We aimed to contribute to understanding the course of COVID-19 and improving clinical strategies. Myocardial depression is completely reversible in patients whose troponin levels were not elevated in the acute phase. Cardiac systolic functions may decrease transiently in the acute phase and improve in the healing process. This change in LVEF may be detected in patients who have severe illness in the acute phase. Further studies are needed to evaluate whether the detection of serum levels of specific cytokines contributes to predicting cardiac involvement and determining treatment strategy.

## Figures and Tables

**Figure:**
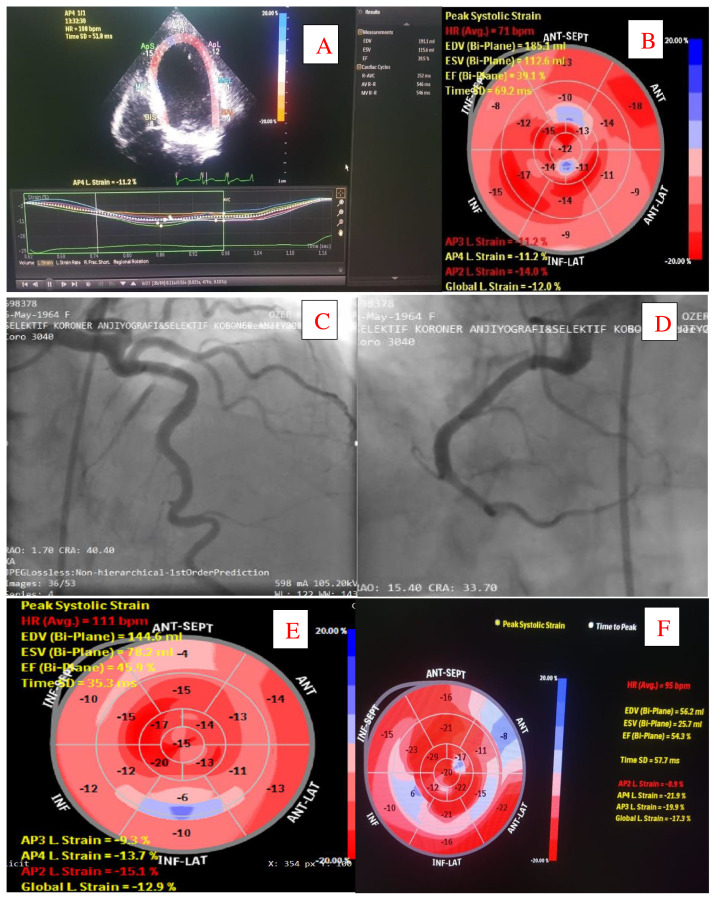
A patient with a transient decrease in systolic function. Echocardiographic imaging showed diffuse left ventricular hypokinesis and left ventricular dysfunction (LVEF= 39.1%) **(A and B)**. She was treated with antiviral drugs (favipiravir), chloroquine, medical treatment for heart failure, and high flow oxygen therapy with progressive clinical stabilization. Normal coronary arteries were detected in coronary angiography **(C and D)**. High-sensitivity troponin T levels were within normal limits in follow-up. Echocardiography follow-up revealed that LVEF was 45.9% on the eighteenth day **( E )** and 54.3% on the thirtieth day **(F)** after clinical improvement begins.

**Table 1:** Baseline characteristics in the first evaluation.

Parameter	All (n = 150)	Transient myocardial dysfunction		p Value
		Decreased LVEF subgroup (n = 15)	Other patients (n = 135)	
Age, mean ± SD, year	46 ± 5	55 ± 9	45 ± 13	0.02
Male, n (%)	78 (52)	8 (53)	69 (51)	0.34
BMI, mean±SD (kg / m^2^)	30 ± 4	34 ± 4	30 ± 6	0.04
Cause of admission, %				
Fatigue, weakness	40	42	39	
Fever	52	57	48	
Dyspnea	20	33	15	
Cough	74	75	60	
Sore throat	42	45	39	
Duration of complaints, (day)	6 ± 3	16 ± 3	5 ± 2	0.01
Smoking, n (%)	24 (16)	2 (13)	22 (16)	0.15
Diabetes mellitus, n (%)	30 (20)	3 (20)	27 (20)	0.82
Hypertension, n (%)	27 (18)	3 (20)	24 (17)	0.22
Asthma, COPD, n (%)	33 (22)	3 (20)	30 (22)	0.78
Malignancy, n (%)	3 (2)	1 (6)	2 (1)	0.10
Illness severity				
Mild illness, n (%)	55 (36)	0	55 (55)	0.01
Moderate illness, n (%)	45 (64)	0	45 (45)	0.01
Severe illness, n (%)	50 (33)	15 (100)	35 (25)	0.01
Patients who need ICU, n (%)	50 (33)	4 (26)	46 (34)	0.09
Heart rate, mean ± SD, bpm	95 ± 10	98 ± 7	94 ± 11	0.06
SBP, mean ± SD, mm Hg	135 ± 10	133 ± 10	136 ± 8	0.21
DBP, mean ± SD, mm Hg	84 ± 4	85 ± 5	84 ± 5	0.30
Laboratory parameters				
WBC mean ± SD (10^9 / L)	7.8 ± 1.3	8.2 ± 3.6	7.7 ± 2.3	0.76
NEU mean ± SD (10^9 / L)	6.0 ± 3.3	6.2 ± 4.3	5.9 ± 3.2	0.66
LYM mean ± SD (10^9 / L)	2.2 ± 1.6	2.3 ± 1.2	2.1 ± 0.6	0.55
CRP mean ± SD (mg / L)	94 ± 10	95 ± 14	93 ± 9	0.10
AST mean ± SD (IU / L)	31 ± 8	26 ± 5	33 ± 8	0.56
ALT mean ± SD (IU / L)	29 ± 6	34 ± 7	27 ± 5	0.44
Blood urea nitrogen mean ± SD (mg / dL)	37 ± 16	47 ± 10	35 ± 15	0.04
Creatinine mean ± SD (mg / dL)	0.88± 0.1	0.96 ± 0.1	0.84 ± 0.15	0.02
Albumin mean ± SD (g / L)	29 ± 16	37.8 ± 11	27.6 ± 15	0.02
Ferritin mean ± SD (pg / L)	450 ± 98	557 ± 110	434 ± 90	0.01
Iron (Fe) mean ± SD (pg / dL)	16 ± 7	17 ± 5	16 ± 6	0.23
UIBC mean ± SD (pg / dL)	199 ± 76	201 ± 55	196 ± 69	0.14
TIBC mean ± SD (pg / dL)	215 ± 95	218 ± 92	212 ± 88	0.43
Ddimer mean ± SD (ng / mL)	525 ± 38	540 ±42	520 ± 31	0.32
Echocardiography				
LVEF (%)	64 ± 6	43 ± 4	61 ± 7	0.01
PABs (mmHg)	25 ± 6	38 ± 8	24 ± 4	0.03
LVEDD (mm)	48 ± 4	55 ± 3	44 ± 2	0.03
LVESD (mm)	32 ± 4	37 ± 2	30 ± 5	0.02
PW (mm)	8 ± 2	7 ± 1	8 ± 1	0.61
LA (mm)	29 ± 3	30 ± 3	28 ± 3	0.48
AR (mm)	26 ± 3	28 ± 2	26 ± 3	0.31
PF (cm / s)	1.6 ± 0.4	1.6 ± 0.6	1.7 ± 0.3	0.46
TAPSE (mm)	18 ± 5	18 ± 3	17 ± 4	0.40

The Mann–Whitney U test was used to compare differences between patients with transient decreased LVEF and other patients.

**Abbreviations:** WBC=White blood cell count, LYM= Lymphocyte, NEU= Neutrophil, AST= Aspartate aminotransferase, ALT= Alanine aminotransferase, CRP= C-reactive protein, LDH=lactate dehydrogenase, TIBC= Total Iron Binding Capacity, UIBC=Unsaturated Iron Binding Capacity, ICU=intensive care unit, COPD= Chronic obstructive pulmonary disease, SBP=Systolic blood pressure, DBP=Diastolic blood pressure, LVEF= left ventricular ejection fraction, PABs=pulmonary systolic artery pressure, LVEDD= left ventricular end-diastolic diameter, LVESD= left ventricular end-systolic diameter, PW=posterior wall thickness at end-diastole, LA=left atrial anteroposterior dimension, AR=aortic root dimension, TAPSE= tricuspid annular plane systolic excursion, PF=pumonary artery flow.

**Table 2:** Medications used.

	Dosage	Duration of use	Number of patients
Favipiravir	day 1: 1600 mg twice daily; days 2–5: 600 mg twice daily	5 days	150
Hydroxychloroquine	days 1–5: 200 mg twice daily (2 × 400 mg loading dose until 31st. of july applied)	5 days	144
Dexamethasone	6 mg daily	10 days	50

**Table 3:** Comparison of the first and the second echocardiographic findings of one hundred fifty patients.

	First echocardiographic evaluation	Second echocardiographic evaluation	p value
LVEF (%)	64 ± 6	60 ± 5	0.52
PABs (mmHg)	25 ± 6	23 ± 5	0.09
LVEDD (mm)	48 ± 4	49 ± 4	0.42
LVESD (mm)	32 ± 4	29 ± 4	0.44
PW (mm)	8 ± 2	8 ± 1	0.65
LA (mm)	29 ± 3	27 ± 3	0.51
AR (mm)	26 ± 3	25 ± 3	0.39
TAPSE (mm)	18 ± 5	24 + 3	0.09
PF (cm / se)	1.6 ± 0.4	1.4 ± 0.5	0.17

LA, PW, and AR in consecutive echocardiograms were analyzed by use of the signed Wilcoxon rank-sum test, and other echocardiographic parameters in consecutive echocardiograms were compared with the paired t test.

**Abbreviations:** LVEF = left ventricular ejection fraction, PABs=pulmonary systolic artery pressure, LVEDD= left ventricular end-diastolic diameter, LVESD= left ventricular end-systolic diameter, PW=posterior wall thickness at end-diastole, LA=left atrial anteroposterior dimension, AR=aortic root dimension, TAPSE= tricuspid annular plane systolic excursion, PF=pumonary artery flow.

**Table 4:** Comparison of mild/moderate and severe illness in the first examination.

	Mild/moderate illness (n = 100)	Severe illness (n = 50)	p value
Age, mean ± SD, year	45 ± 12	54 ± 10	<0.01
BMI, mean ± SD (kg / m^2^)	30 ± 6	35 ± 5	0.01
Duration of complaints (day)	5 ± 4	13 ± 3	0.02
Echocardiography			
LVEF (%)	59 ± 6	50 ± 6	0.03
PABs (mmHg)	20 ± 6	24 ± 8	0.05
LVEDD (mm)	48 ± 4	52 ± 4	0.43
LVESD (mm)	31 ± 4	35 ± 4	0.04
PW (mm)	8 ± 1	7 ± 1	0.62
LA (mm)	29 ± 3	27 ± 3	0.51
AR (mm)	26 ± 3	25 ± 3	0.31
PF (cm/s)	1.5 ± 0.5	1.6 ± 0.4	0.52
TAPSE (mm)	20 ± 3	19 ± 3	0.09

The Mann-Whitney U test was used for LA and PW. The independent samples t-test was used for all other data.

**Abbreviations:** LVEF= left ventricular ejection fraction, LVEDD= left ventricular end-diastolic diameter, LVESD= left ventricular end-systolic diameter, BMI= body mass index, PW=posterior wall thickness at end-diastole, LA=left atrial anteroposterior dimension, AR=aortic root dimension, TAPSE=tricuspid annular plane systolic excursion, PF=pumonary artery flow, PABs=pulmonary systolic artery pressure

**Table 5:** Comparison of the first and the second echocardiographic findings of fifteen patients with transient decreased LVEF.

	First echocardiographic evaluation	Second echocardiographic evaluation	p value
LVEF (%)	43 ± 4	55 ± 2	0.01
PABs (mmHg)	38 ± 8	23 ± 5	0.09
LVEDD (mm)	55 ± 3	49 ± 4	0.04
LVESD (mm)	37 ± 2	29 ± 4	0.05
PW (mm)	7 ± 1	7 ± 1	0.61
LA (mm)	30 ± 3	28 ± 3	0.09
AR (mm)	28 ± 2	28 ± 3	0.32
PF (cm / sec)	1.6 ± 0.6	1.4 ± 0.5	0.13
TAPSE (mm)	18 ± 3	23 + 3	0.09

All data in consecutive echocardiograms were analyzed by use of the signed Wilcoxon rank-sum test.

**Abbreviations:** LVEF= left ventricular ejection fraction, PABs=pulmonary systolic artery pressure, LVEDD= left ventricular end-diastolic diameter, LVESD= left ventricular end-systolic diameter, PW=posterior wall thickness at end-diastole, LA=left atrial anteroposterior dimension, AR=aortic root dimension, TAPSE= tricuspid annular plane systolic excursion, PF=pumonary artery flow.
